# The Biarzo case in northern Italy: is the temporal dynamic of swine mitochondrial DNA lineages in Europe related to domestication?

**DOI:** 10.1038/srep16514

**Published:** 2015-11-09

**Authors:** Stefania Vai, Sibelle Torres Vilaça, Matteo Romandini, Andrea Benazzo, Paola Visentini, Marta Modolo, Marco Bertolini, Peggy MacQueen, Jeremy Austin, Alan Cooper, David Caramelli, Martina Lari, Giorgio Bertorelle

**Affiliations:** 1 Dipartimento di Biologia, Università di Firenze, Firenze, Italy; 2 Dipartimento di Scienze della Vita e Biotecnologie, Università di Ferrara, Ferrara, Italy; 3 Dipartimento di Studi Umanistici, Sezione di Scienze Preistoriche e Antropologiche, Università di Ferrara, Ferrara, Italy; 4 Museo Friulano di Storia Naturale, Udine, Italy; 5 Australian Centre for Ancient DNA, University of Adelaide, Adelaide, Australia

## Abstract

Genetically-based reconstructions of the history of pig domestication in Europe are based on two major pillars: 1) the temporal changes of mitochondrial DNA lineages are related to domestication; 2) Near Eastern haplotypes which appeared and then disappeared in some sites across Europe are genetic markers of the first Near Eastern domestic pigs. We typed a small but informative fragment of the mitochondrial DNA in 23 *Sus scrofa* samples from a site in north eastern Italy (Biarzo shelter) which provides a continuous record across a ≈6,000 year time frame from the Upper Palaeolithic to the Neolithic. We additionally carried out several radiocarbon dating. We found that a rapid mitochondrial DNA turnover occurred during the Mesolithic, suggesting that substantial changes in the composition of pig mitochondrial lineages can occur naturally across few millennia independently of domestication processes. Moreover, so-called Near Eastern haplotypes were present here at least two millennia before the arrival of Neolithic package in the same area. Consequently, we recommend a re-evaluation of the previous idea that Neolithic farmers introduced pigs domesticated in the Near East, and that Mesolithic communities acquired domestic pigs via cultural exchanges, to include the possibility of a more parsimonious hypothesis of local domestication in Europe.

The transition from foraging and hunting to farming and herding is a significant process in human history. Archaeological evidence suggests that early domestication events took place in the Fertile Crescent approximately 11,000 years before present (BP)^1^. Neolithic technologies were then introduced into Europe starting from ca. 8,000 BP by colonists along two trajectories, the Mediterranean and the Danube-Balkan route^1^,^2^.

The history of domestic sheep and goat in Europe is relatively simple, since wild forms were not present and they descend only from animals domesticated elsewhere. The scenario is clearly different for cattle and pigs, since the aurochs was, and the wild boar still is, widely distributed in Europe. Local domestication processes and/or admixture events between the imported domestic form and the local wild animals cannot be excluded *a priori*. In general, genetic data have provided important contributions to domestication studies, using both modern samples and ancient Neolithic and pre-Neolithic remains^3^,^4^,^5^,^6^,^7^,^8^,^9^. The current genetic reconstruction of *Sus scrofa* domestication in Europe based on mitochondrial DNA (mtDNA) sequences claims that Near Eastern domesticated pigs were introduced between 7,500–6,000 BP^10^,^11^,^12^. A few thousand years later this Near Eastern ancestry of European pigs disappeared due to extensive introgression with local wild boars or with locally domesticated pigs. This conclusion is based on the presence in early domestic pig samples of mtDNA sequences which are found today only in Near Eastern wild boars (lineages Y1, Y2, Arm1T, belonging to the NE2 clade^11^,^12^,^13^,^14^,^15^,^16^), and on the absence of such lineages both in pre-Neolithic samples and in all European samples dated from 6,000 BP to the present. Under the same hypothesis, Near Eastern lineages later (2,000–3,000 BP) disappeared in Near Eastern pigs too, as a consequence of commercial trades that imported European breeds in those areas^10^,^11^. This introduction, followed by hybridization between domestic and wild forms, possibly favoured the extinction of local wild lineages in Israel^17^.

Friuli is a north eastern Italian region connecting the Italian peninsula to the Balkans. Its Neolithization was affected by the high density of Mesolithic Castelnovian communities that favoured a longer co-existence of Mesolithic and Neolithic economic practices and populations. The continuity of indigenous populations alongside new Neolithic groups appears more pronounced in Italy than elsewhere in the Mediterranean^18^,^19^, and domesticated fauna has been sometimes associated to Mesolithic contexts^20^. A similar pattern is observed along the eastern coasts of the northern Adriatic, in Istria and Dalmatia^21^,^22^. Clear evidence of such a process, however, is missing for the Italian peninsula, and is very scant and controversial for other European areas^12^,^13^,^14^,^15^.

The Biarzo shelter is located along the Natisone river in Friuli, a north eastern Italian region (Fig. S1). The geographic position, south of the Alps and in a natural ecological corridor joining Italy and the Balkans, appears particularly favourable for faunal migrations and cultural exchanges. Biarzo shelter, excavated from 1982 to 1984, is the only northern Italy site with a continuous stratigraphy of six stratigraphic units (hereafter, US) from the Upper Palaeolithic, through the Mesolithic, until the Neolithic and the Bronze Age (Fig. 1). While stratigraphy for the lower units is clear, in the upper levels there is evidence of some mixing of Late Mesolithic Castelnovian and Early Neolithic materials in US 3A, most likely caused by later Neolithic frequentation^23^,^24^. Only two radiocarbon dates on charcoal were available prior to our investigation: US 5 yielded a date fully consistent with Late Epigravettian (around 13,000 years ago) while the calibrated range for the layer US 3A (where artefacts attributed to Late Mesolithic Castelnovian and Early Neolithic were found) is very large and supports also a Late Neolithic age (Table S1)^25^,^26^. Additional information on the site and the artefacts found in the different layers is reported in the legend of Figure S1.

This site is a *unicum* in Italy for the presence of *Sus scrofa* as the most represented species in all the stratigraphic levels^27^,^28^. Archaeozoological data cannot distinguish between pigs and wild boars in early Neolithic times, since shape and dimensional differences appear only in later stages. However, indirect evidence such as sex and age structure can discriminate between wild animals and managed herds^29^. In Biarzo, both the slight increased frequency of young *Sus scrofa* individuals in the upper levels (66% in US 3A versus 50% in US 4+3B), and the presence of *Bos taurus* (USs 3A and 2) and Ovicaprinae (US 2), suggests that this site witnessed the transition between wild and domestic boars. The Biarzo shelter is therefore an optimal candidate to study the dynamic of the boars consumed across a large temporal frame of ≈6,000 year encompassing the Neolithic cultural transition. Our approach was to analyse a highly informative fragment of the mitochondrial DNA in pig remains of different ages sampled from this site, in order to investigate the temporal distribution of lineages, and possibly to associate this with either natural or domestication-related processes.

## Results

AMS dating of five pig remains from US 3A and US 3B produced pre-Neolithic dates. The most ancient dates were obtained from the two samples of US 3B (5012 and 5013) and are compatible with Sauveterrian Mesolithic culture; two samples of US 3A are in the range of Sauveterrian Mesolithic (3273 and F3479), while a third sample gave a date compatible with Castelnovian Mesolithic (3760). The detailed results of AMS dating are reported in Table 1. All these dates support strongly the idea that US 3A is Mesolithic, despite some contamination from the upper US 2 level that produced a dubious cultural association. The previous charcoal date of US 3A (Table S1) that produced a very large confidence interval with a lower limit of 5,856 years, is probably inaccurate. With the exception of the single individual from Neolithic US 2, we therefore conclude that all the samples we analysed can be attributed to pre-Neolithic (and thus pre-domestication) times.

Contamination from modern pig DNA in the laboratory and in the reagents can be confidently excluded since only one out 54 negative PCR controls gave an amplification product, while no amplification results were obtained from negative extraction controls. In addition, about three quarters of the sequences motifs we found in the samples from Biarzo shelter belong to haplotypes that are not observed today in modern European breeds.

We obtained sequences from 24 different individuals, while five samples gave no amplification results and were therefore discarded. The sample 5012 gave ambiguous sequence results across different PCRs from different extracts, while the sample 921 + 922 gave only one amplification product. In order to confirm their genetic assignment, a subset of these two samples was sent to the Australian Centre for Ancient DNA (ACAD) along with two other randomly selected samples (2338, 1138). In the replication at ACAD the sequences of the samples 2338 and 1138 were confirmed to present the same haplotype obtained in all the amplicons sequenced in Florence, while the samples 5012 and 921 + 922 gave no amplification products. Considering the inconsistency in the results, the sequences of the two latter samples were then excluded from the analyses (Table S2), resulting in 22 confidently different individuals. In addition, we obtained positive amplification and sequencing results in all three additional samples for which the attribution to further individuals was uncertain (see Table S4). One of these samples, 2806, gave a sequence that was different from the sequence obtained from the other anatomical element putatively belonging to the same individual (3273). For this reason the sample 2806, belonging to a new different individual, was included in the subsequent analyses, while the other two samples, 2320 and 2106, were discarded because they both had the same sequences of the possible duplicates.

Globally 23 individuals produced positive and reproducible results (Table S3). Consensus sequences were deposited in GenBank, with accession numbers KT949925-KT949947. As expected, an increasing fraction of positive results were obtained as the age of the specimens decreased: eight out of 14 from US 5, six out of eight from US 4, two out of three from US 3B, six out of six from US 3A and one out of one from US 2.

**Figure 2:**
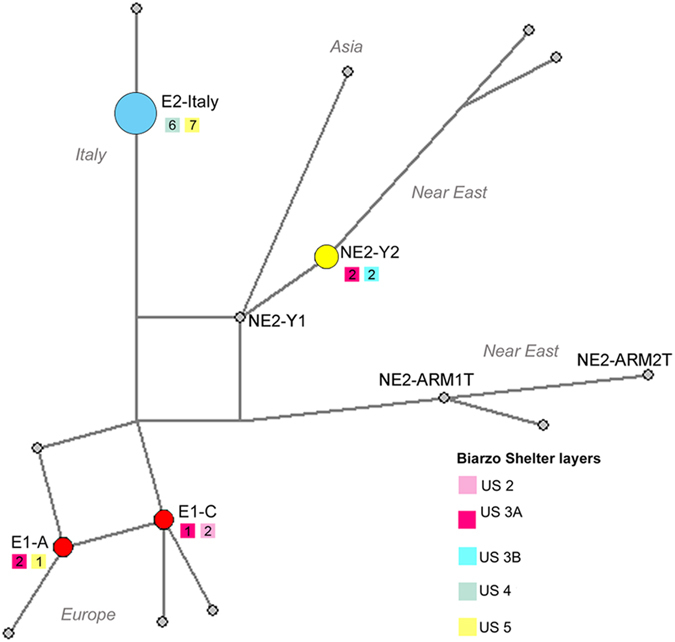
Median joining network of Biarzo shelter mtDNA results integrated with previously reported ancient and modern data. Data obtained in this study are shown in different colours (related also to Fig. 3), data from other studies^10^,^11^ are in grey; haplotype frequencies were indicated only for the samples from Biarzo shelter. The coloured squares below the haplotype names indicate the stratigraphic layers and the number of individuals in which each haplotype was found (colours according to Fig. 1). Association between the haplotypes and their putative geographic origin as inferred in^10^ was also indicated.

Five different haplotypes were found - all matching sequences previously described in the literature. In particular, considering the haplotype network reconstructed including significant haplotypes from the major clades (Fig. 2), we were able to classify each individual from Biarzo into haplogroups commonly associated to specific geographic regions (Table 2).

The most relevant result of our study is the change of frequencies that occurred at the Biarzo site for three major pig mtDNA lineages. The major lineage turnover can be dated back to the Mesolithic, and implied the extinction of a typical Italian clade and the appearance of a haplogroup commonly associated to wild boars from Near Eastern areas and commonly used as a marker of domestication. We now describe these results in more detail.

### mtDNA haplotype turnover

**Figure 3:**
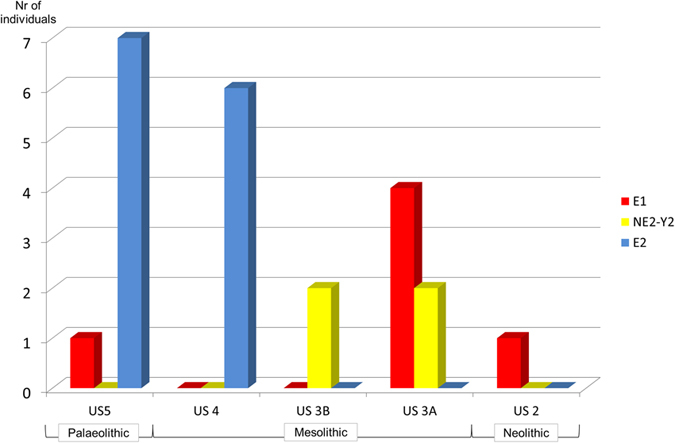
Temporal distribution of mitochondrial haplotypes in Biarzo. For each stratigraphic unit (x axis) the number of samples with a specific mitochondrial haplotype is reported.

Almost all the samples from US 5 (7 out of 8) and all the six samples from US 4, i.e. from layers that can be safely considered on the basis of the cultural context and the AMS dating of upper layers to be at least 10,000 years old, belong to a haplogroup called E2. This lineage, known also as “the Italian clade”, has been previously found only in ancient Italian and Croatian specimens^10^, but today is observed only in central and southern Italian and Sardinian natural (i.e., not reintroduced) wild boar populations, and never in domestic pigs^30^,^31^,^32^,^33^. E2 sequences completely disappeared from the Biarzo site starting from level US 3B (Table 2 and Fig. 3), when NE2-Y2 initially appeared followed by E1 in US 3A. NE2-Y2 is found today only in Near Eastern wild boars, and E1 is the pan-European mtDNA clade both in wild and domestic pigs. This result supports the view that during the Mesolithic, probably in a short period of time from some hundred to a few thousand years, a dramatic replacement occurred in the genetic composition of the wild boars eaten by the Biarzo inhabitants.

### Near Eastern sequences in the Mesolithic layers?

Particular attention is required for the temporal distribution of NE2-Y2 lineage in our data, due to the fact that NE2 clade is found today only in the Near East (as the name suggests) and its presence in several ancient Central and Western European specimens was associated with the arrival of the Neolithic culture^10^,^12^.

Five specimens from at least four different individuals had a NE2-Y2 sequence, and the radiocarbon dates directly or indirectly exclude their attribution to domestic Neolithic pigs. Three specimens from at least two different individuals came from layer US 3B, which is clearly associated to a Mesolithic Sauveterrian culture. One of these bone fragments (5013, see Table 1) was directly dated to 9,890–9,605 years ago. From the same layer, a fragment for which genetic analysis was unsuccessful (5012) provided an even earlier date, between 10,235 and 10,480 years ago. The other two NE2-Y2 sequences were obtained from two samples collected in the US 3A, very likely associated to Castelnovian and Sauveterrian Mesolithic culture. One of them was directly dated at 9,530–9,555 years ago.

The situation in Biarzo is thus conflicting with the idea that haplotypes observed today in the Near East, when found in ancient samples, indicate the arrival of domestic pigs. The dates we directly obtained for two NE-Y2 samples clearly fall in the range of the Sauveterrian Mesolithic, pre-dating by at least two millennia the arrival of Neolithic package in the same area^34^, and the other dates inferred for NE-Y2 samples are associated to pre-Neolithic layers.

## Discussion

The motivation of this study was to reconstruct the temporal dynamics of mtDNA pig lineages along a continuous temporal framework of several millennia in a single archaeological site. As this site is located in a crossroad area between Italy, the Balkans and Central Europe we believed that the Biarzo case provides a unique opportunity to document the impact of domestication on wild and domestic pig consumption across the Neolithic transition, while at the same time avoiding the confounding influence of geographical variation on the temporal pattern.

After re-consideration of the age of some of the samples we analysed, which were initially attributed to a Neolithic layer but subsequently AMS dated to 9 to 10 thousand years ago, we can now say that we missed our main goal, but we probably accomplished a more important task: we showed that the two major pillars of the previous genetically-based reconstructions of the history of pig domestication in Europe^10^,^12^ are unstable.

The general hypothesis based on mtDNA on pig domestication, formulated by Larson in two seminal papers^10^,^35^, affirms that pigs domesticated in the Near East were introduced into Europe with the Neolithization of the continent, but that soon after the genetic legacy of these pigs and their descendants were lost due to constant hybridization with European wild boars, replacement by pigs domesticated in Europe, or both. This hypothesis is based on two pillars: 1) temporal changes of mtDNA lineages in Europe are related to domestication, and 2) NE-Y mtDNA lineages appearing and then disappearing in some sites in Europe are genetic markers of Near Eastern domestic pigs. Clearly, our main results shake these pillars. We found in fact that large changes in the composition of pig mtDNA lineages can occur in a single site across few millennia, independently of the domestication process, and that NE-Y mtDNA lineages were present in Europe much earlier than the arrival of Neolithic breeders or culture.

Starting from at least 9800 BP, wild boar belonging to typical Italian E2 clade, that represented almost the only meat source for Biarzo Paleolithic and early Mesolithic communities, was rapidly and completely replaced by animals bearing very different mitochondrial haplotypes: E1 and NE-Y2. A possible explanation for this haplotype turnover is a natural replacement of prey composition due to migrations/dispersal of wild boar individuals from different populations, possibly favoured by climatic variations at the end of the last glaciation^36^,^37^. Along with this hypothesis, Vila**ç**a *et al.* (2014)^32^ suggest a post-glacial expansion from Italy of E1 bearing groups, but for the Biarzo area we should also speculate that the replacement of E2 had involved also wild boars with high frequency of NE-Y2 haplotypes. In principle, we cannot exclude that the abrupt genetic turnover we observe is due to a change of preferred boars hunted from a population with stable genetic composition. This hypothesis requires an association between a morphological trait of interest for the Mesolithic hunters (e.g., the size) and a specific mtDNA haplotype (e.g., E1 or NE-Y vs. E2). Such a correlation between traits and mtDNA haplotypes has been reported in some marine molluscs^38^,^39^, but not in mammals so far. A preliminary analysis based on the weight of 34 E1 and E2 wild boars from the same area in Sardinia excluded this association (M. Scandura, personal communication), but more data are clearly needed. At this stage we can only conservatively exclude the hypothesis of change in hunted targets in the Mesolithic, and record that a mtDNA turnover occurred in the wild boar population.

Concerning the appearance and rapid disappearance of NE haplotypes in other European sites, two important aspects should be mentioned. First, pre-Neolithic wild boar samples in Central and Northern Europe are numerically and geographically limited, and it seems therefore premature to exclude the presence of NE haplotypes (see for example the recent paper by Evin *et al.* 2015^40^), possibly also at high frequencies in some areas (as in Biarzo), before domestication. Second, most of the NE sequences found in Europe belong to the NE-Y1 haplogroup, and only a small fraction shows exactly the same sequence of the haplotype found in Biarzo, NE-Y2, that differ from NE-Y1 for a single mutation (an indel). We cannot therefore exclude that NE-Y2 haplotypes were present in Europe since 10,000 years ago, and NE-Y1 haplotypes arrived in the Neolithic from the Middle East. This possibility appears however rather implausible and not parsimonious.

In conclusion, we suggest that the hypotheses based on mtDNA data that Neolithic farmers introduced into western Europe pigs domesticated in the Near East, and that Mesolithic hunter-gatherer communities acquired domestic pigs from them via cultural exchanges^10^,^12^, should be reconsidered. Our working hypothesis, to be verified with additional data from more pre-Neolithic sites and, possibly, more genomic markers, is that NE haplotypes were present in Europe at least two millennia before the arrival of the Neolithic package, at low frequencies in some areas for which pre-Neolithic samples have been typed without finding them, or at larger frequencies (as in Biarzo for NE-Y2) in some yet un-sampled areas. If this is true, the history of modern European pig breeds might be simplified to a continuous process of local domestication without the need of a Near Eastern wave of introgression.

## Methods

### Samples analyzed

On the basis of the Minimum Number of Individuals (MNI)^41^, we selected for DNA analysis 29 *Sus scrofa* samples which had been recovered from five different stratigraphic units and represented three different archaeological contexts. We analysed 14 individuals from the US 5 (Upper Palaeolithic, Late Epigravettian culture), 9 individuals from US 4 and US 3B (Early Mesolithic, Sauveterrian culture), 5 individuals from US 3A (Early Neolithic/Late Mesolithic, Castelnovian culture) and 1 individual from US 2 (Middle Neolithic). We selected only teeth, single or included in fragments of bone. We also included three additional samples for which the attribution to further different individuals could not be ascertained by archaeozoological methods, in order to verify their individuality by genetic assignment (Table S4).

### Radiocarbon Dating

We performed Accelerator Mass Spectrometry (AMS) dating on five pig remains from US 3B and 3A to better resolve the chronology of the site and to precisely date the appearance of specific haplotypes (Table 1). Due to the very small size of the analysed samples, it was possible to make direct dating on only one of the individuals genetically typed (3273 from US 3A). We therefore also selected additional remains not included in the Minimum Number of Individuals (3760 and F3479 from US 3A; 5013 from US 3B) or for which genetic typing was unsuccessful (5012 form US 3B). The haplotypes of two additional bone fragments were determined (F3479 from US 3A and 5013 from US 3B) and the sequences were used for dating the appearance of the specific haplotypes but not for the phylogenetic analyses. Dates were obtained from the Beta Analytic radiocarbon dating laboratory (Miami, FL, USA), and were calibrated according to Talma, A. S., Vogel, J. C.^42^, using the INTCAL13 atmospheric curve^43^.

### General equipment

Standard criteria for the analysis of ancient samples were followed^44^,^45^ and multiple measures were undertaken to exclude contamination by exogenous DNA. When dealing with ancient samples the risk of false positive results due to contamination from modern DNA is a crucial issue. For domestic species especially, a significant amount of DNA is present in the environment and previous studies reported the sporadic presence of DNA from cow, pig and chicken in PCR reagents^46^. DNA extractions and PCR set up were carried out in “pre-PCR” clean-rooms irradiated by UV after each work session and physically separated from the areas in which PCR amplification and post-PCR analysis were conducted. Disposable face masks, gloves, over-shoes and laboratory coats were worn during the experiments and were changed frequently. All benches and rooms were routinely treated with bleach and UV-light. Extraction and amplification blanks were used as negative controls in each reaction. In addition, after completing all the molecular procedures, a subset of four samples were sent to the Australian Centre for Ancient DNA in Adelaide (Australia) for blind replication.

### Samples preparation and DNA extraction

In the clean-room of the Florence lab, a thin external layer of each tooth was removed using a dental device (Marathon Multi 600) with disposable dental bur, rotating at minimum speed (1000 rpm). Each sample was then UV-irradiated in a cross-linker (254 nm wavelength) for 45 minutes and subsequently ground into a fine powder with the same dental device. DNA was extracted twice, at different times, with two different extraction methods as described in Caramelli *et al.*^47^ and Rohland & Hofreiter^48^.

### DNA amplification

Two μl of the extracted DNA were amplified using a single primer pair, named ANC-F1/ANC-R1, that amplify a ≈128 bp long fragment of pig mtDNA^49^. PCR conditions are described in Mona *et al.*^50^. Each extract was amplified at least twice and in independent PCR reactions.

### Cloning and sequencing

PCR products were cloned using TOPO TA Cloning Kit (Invitrogen) according to the manufacturer’s instructions. Screening of white recombinant colonies was accomplished by PCR, as described in^50^. After purification (Microcon PCR, Amicon), each clone was cycle-sequenced following the BigDye Terminator kit (Applied Biosystems) supplier’s instructions and the sequence was determined using an Applied BioSystems 3100 DNA sequencer. For each amplification product we sequenced at least five clones in order to determine a confident consensus sequence.

### Sample replication at Australian Centre for Ancient DNA (ACAD), Adelaide

On the basis of the results obtained, four samples were selected for blind replication at the Australian Centre for Ancient DNA (ACAD) in Adelaide. All pre-PCR work was performed in a dedicated ancient DNA laboratory geographically separated (by 1.5 km) from post-PCR and other molecular biology laboratories at the Australian Centre for Ancient DNA, University of Adelaide, South Australia. No contemporary pig DNA had ever been present in the pre-PCR laboratory. The ancient DNA facility includes high-efficiency particulate air-filtered positive air pressure with one-way air flow, overhead ultraviolet (UV) lights, individual work-rooms, the use of dead-air glove boxes with internal UV lights for DNA extractions and PCR set-up, regular decontamination of all work areas and equipment with sodium hypochlorite, personal protective equipment including full body suit, face mask, face shield, boots and triple-gloving and strict one-way movement of personnel (shower > freshly laundered clothes > ancient DNA laboratory > post-PCR laboratory). A negative extraction control was included with all DNA extractions. DNA was extracted from pre-powdered tooth samples using a modified silica-based method^51^ designed to maximize recovery of PCR-amplifiable DNA from ancient bone and tooth specimens while minimizing coextraction of PCR inhibitors. The same primer pair was used for DNA amplification, amplicon purification and Sanger sequencing as described by Austin *et al.*^52^.

### Haplotype determination and phylogenetic analyses

The consensus sequences, together with a set of sequences representative of ancient and modern samples^10^, were aligned using ClustalX^53^. Haplotypes were then assigned using the nomenclature proposed in Larson *et al.*^10^ and Ottoni *et al.*^11^. A median-joining network of the Biarzo samples and the reference data set was then constructed by means of Network 4.1^54^,^55^; transversions were weighted 2.0 and indels two times as high as transitions.

## Additional Information

**How to cite this article**: Vai, S. *et al.* The Biarzo case in northern Italy: is the temporal dynamic of swine mitochondrial DNA lineages in Europe related to domestication? *Sci. Rep.* **5**, 16514; doi: 10.1038/srep16514 (2015).

## Supplementary Material

Supplementary Information

## Figures and Tables

**Figure 1:**
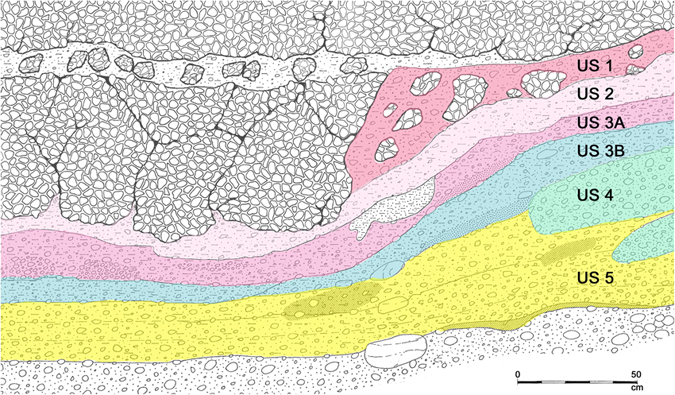
Stratigraphy of Biarzo shelter. Cultural attributions of the stratigraphic units (USs) are: US 5 Late Epigravettian; US 4 and US 3B Sauveterrian Mesolithic; US 3A Early Neolithic/Castelnovian Mesolithic; US 2 Middle Neolithic. Approximately dates of these material cultures in the area of Biarzo shelter are: Late Epigravettian 16,000–11,500 Cal BP; Sauveterrian Mesolithic 11,500–8,800 Cal BP; Castelnovian Mesolithic from 8,800 Cal BP; Early Neolithic from 7,600 Cal BP; Middle Neolithic from 7,200 Cal BP. (Original drawing granted by Museo Friulano di Storia Naturale)

**Table 1:** AMS radiocarbon dates on *Sus scrofa* samples.

US	Sample	Haplotype	Lab Code	Conventional Radiocarbon Age	2 Sigma Calibrated Results 95% Probability
3A	3760	n.a.	Beta – 396322	7950 + /−30 BP	Cal BP 8990 to 8640
3A	F3479	E1-C	Beta – 404755	8280 + /−30 BP	Cal BP 9405 to 9340 and Cal BP 9330 to 9235 and Cal BP 9225 to 9200 and Cal BP 9180 to 9135
3A	3273	Y2	Beta – 396319	8600 + /−30 BP	Cal BP 9555 to 9530
3B	5013	Y2	Beta – 396321	8750 + /−30 BP	Cal BP 9890 to 9605
3B	5012	n.a	Beta – 396320	9170 + /−40 BP	Cal BP 10480 to 10465 and Cal BP 10430 to 10235

**Table 2:** Haplotype designations of the 23 pig samples from Biarzo shelter.

Sample code	US	Haplotype	Putative geographic region of origin
1	2	E1-C	Europe
3740	3A	E1-C	Europe
3810	3A	NE2-Y2	Near East
4519	3A	E1-A	Europe
3377	3A	E1-A	Europe
3273	3A	NE2-Y2	Near East
2806	3A	E1-C	Europe
3310	3B	NE2-Y2	Near East
4374	3B	NE2-Y2	Near East
2095	4	E2	Italy
2318	4	E2	Italy
2338	4	E2	Italy
1941	4	E2	Italy
1938	4	E2	Italy
2222	4	E2	Italy
1093	5	E2	Italy
4793	5	E2	Italy
256	5	E1-A	Europe
5	5	E2	Italy
920	5	E2	Italy
1138	5	E2	Italy
195	5	E2	Italy
2986	5	E2	Italy
