# Supplementary material for: The Biarzo case in northern Italy: is the temporal dynamic of swine mitochondrial DNA lineages in Europe related to domestication?
Source: Sci Rep. 2015 Nov 9;5:16514. doi: 10.1038/srep16514 (PMC4637886; doi:10.1038/srep16514)
Supplement: Supplementary Information [file srep16514-s1.pdf]

Supplementary Materials

The Biarzo case in northern Italy: is the temporal dynamic of swine mitochondrial DNA lineages in Europe related to domestication?

Stefania Vai[1], Sibelle Torres Vilaça[2], Matteo Romandini[3], Andrea Benazzo[2], Paola Visentini[4], Marta Modolo[3], Marco Bertolini[3], Peggy MacQueen[5], Jeremy Austin[5], Alan Cooper[5], David Caramelli[1], Martina Lari[1*], Giorgio Bertorelle[2].

1 Dipartimento di Biologia, Università di Firenze, Firenze, Italy

2 Dipartimento di Scienze della Vita e Biotecnologie, Università di Ferrara, Ferrara, Italy

3 Dipartimento di Studi Umanistici, Sezione di Scienze Preistoriche e Antropologiche, Università di Ferrara, Ferrara, Italy

4 Museo Friulano di Storia Naturale, Udine, Italy

5 Australian Centre for Ancient DNA, University of Adelaide, Adelaide, Australia

* Corresponding author: Martina Lari

e-mail: martina.lari@unifi.it; phone: +39 055 2757740; fax: +39 055 2757753

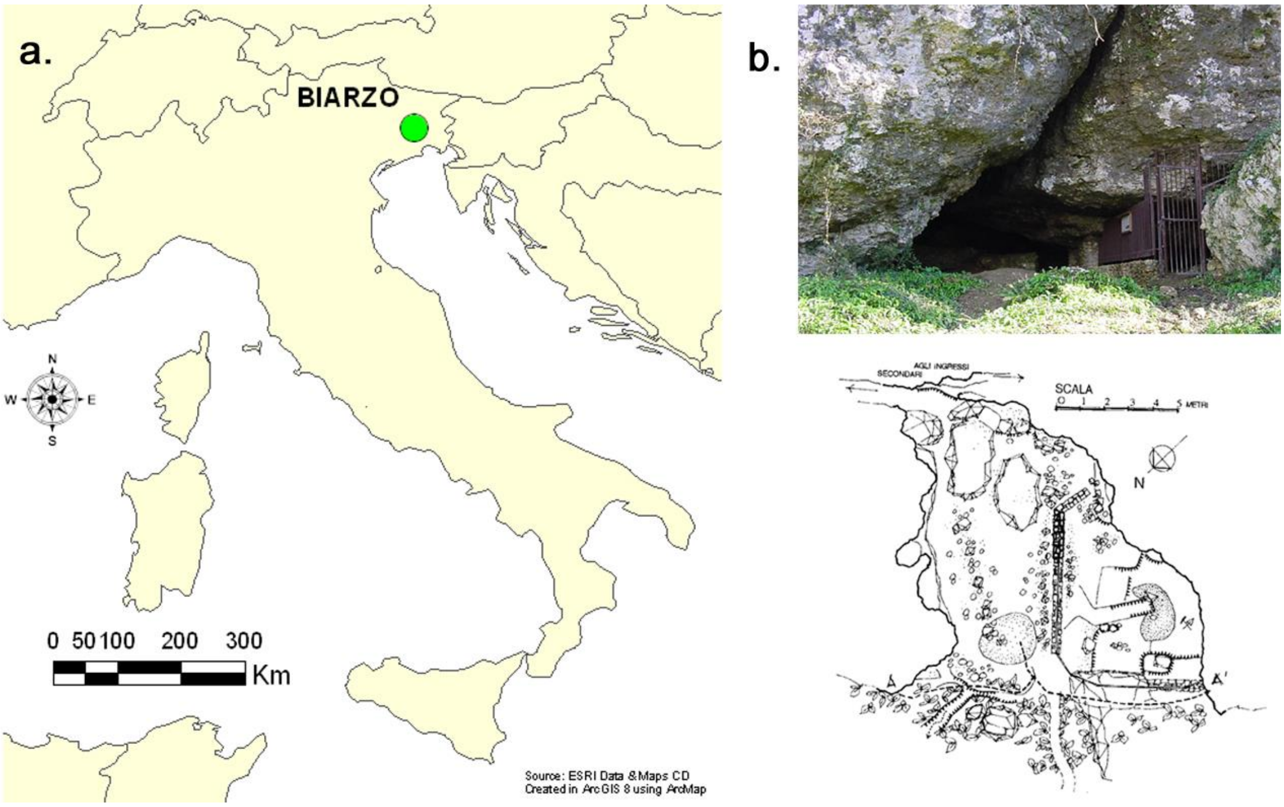

Figure S1: The archeological site of Biarzo Shelter.

a. Maps showing Biarzo Shelter position. The map was created in ArcGIS version 8 using ArcMap**. b. Picture of the entrance and planimetry of the site.** Biarzo Shelter is located in the Natisone River valley (Udine, Friuli Venezia Giulia, Italy). The lithic industry from US5 is mainly characterized by scrapers, in particular the small circular ones (thumbnails scrapers), burins, truncations, becks and backed blades [1]. Functional analysis carried out on the lithic material has revealed that the processing of animal material (e.g. hunting, hide treatment, bone working) represented the main activity carried out at the site [2]. The lithic industry from layers 4 and 3B represents a typical Sauveterrian industry, characterized by burins, backed points, truncations, geometrics such as triangles (comparable to the triangles of Montclus) microburins, tools with backed retouch, which is comparable to the upper part of layer AC of Riparo Romagnano in the Adige Valley [1]. In layer 3A some trapezes, which are typical of the later Castelnovian phase, have also been recovered, whereas in the upper part of layer 3A Impresso potsherds have been found. Geo-archaeological data report the erosion of the more superficial part of layer 3A, where materials from layers 2 and 1 have been found [1]. The picture and the drawing were granted by Museo Friulano di Storia Naturale.

[1]Bressan, F., Cremaschi, M. & Guerreschi, A. Nuovi dati sulla preistoria in Fruili: il Riparo di Biarzo (scavi 1982), S. Pietro al Natisone (Udine). *Gortania. Atti Mus. Fruil. St. Nat.* **4**, 65-86 (1982). [2]Ziggiotti, S. Il Riparo di Biarzo (S. Pietro al Natisone, Udine). Contributo alla ricostruzione della funzione dell'insediamento attraverso lo studio delle tracce d'uso. *Gortania* **29**, 55-72 (2007)

Table S1: Previously available radiocarbon dating for stratigraphic units (US)

US	Sample	Lab Code	Conventional Radiocarbon Age	2 Sigma Calibrated Results *(OxCal v4.1.7, Bronk Ramsey 2013)*
3A	charcoal	R1S51	5,600 +/- 300 BP	Cal BP 7161 to 5856 at 93.5% probability
5	charcoal	R1850	11,100 +/- 125 BP	Cal BP 13177 to 12723 at 95.4% probability

Table S2: Summary results of the replication test

**sample**	**n° of positive amplification Florence**	**concordance of sequencing results Florence**	**postive ampification ACAD**	**concordance of sequencing results Florence/ACAD**
**5012**	3	no	no	n.a.
**2338**	4	yes	yes	yes (Italian clade)
**1138**	4	yes	yes	yes (Italian clade)
**921+922**	1	n.a.	no	n.a.

Table S3: DNA sequences of clones for each sample (primers are excluded). Above of each sample the reference sequence (GenBank: AJ002189) and the numbering of the nucleotide positions are reported. Nucleotides identical to the reference are indicated by dots in the clones. Clones are identified by a code composed by sample name, number of extraction and PCR identification letter. At the end of the table are reported the consensus sequences of two additional samples selected for dating for which cloning were not performed.

```
           1111111111111111111111111111111111111111111111111111111111 1111111111111111
           5555555555555555555555555555555555555555555555555555555555 5555555555555555
           5555555555555555555555555555555555555555555555555555555555 5555555555555555
           2222222222333333333344444444445555555555666666666677777777 7788888888889999
           0123456789012345678901234567890123456789012345678901234567 8901234567890123

ref        TTGCGCACAAACATACAAATATGTGACCCCAAAAATTTTACCATTGAAAACCAAAAAA TCTAATATACTATAAC

1_1_a      ..........................................................-................
1_1_a      ..........................................................-................
1_1_a      ..........................................................-................
1_1_a      ..........................................................-................
1_1_a      ..........................................................-................
1_1_a      ..........................................................-................
1_1_b      ..........................................................-................
1_1_b      ..........................................................-................
1_1_b      ..........................................................-................
1_1_b      ..........................................................-................
1_1_b      ..........................................................-................
1_2_a      ..........................................................A................
1_2_a      ..........................................................A................
1_2_a      ..........................................................A................
1_2_a      ..........................................................-................
1_2_a      ..........................................................-................
1_2_b      ..........................................................-................
1_2_b      ..........................................................-................
1_2_b      ..........................................................-................
1_2_b      ..........................................................-................
1_2_b      ..........................................................-................

consensus:
1          TTGCGCACAAACATACAAATATGTGACCCCAAAAATTTAACCATTGAAAACCAAAAAA-TCTAATATACTATAAC


           11111111111111111111111111111111111111111111111111111111111111111111111111
           55555555555555555555555555555555555555555555555555555555555555555555555555
           55555555555555555555555555555555555555555555555555555555555555555555555555
           22222222223333333333444444444455555555556666666666777777777788888888889999
           01234567890123456789012345678901234567890123456789012345678901234567890123

ref        TTGCGCACAAACATACAAATATGTGACCCCAAAAATTTTACCATTGAAAACCAAAAAATCTAATATACTATAAC

3740_1_a   ...........T..............................................................
3740_1_a   ...........T..............................................................
3740_1_a   ..........................................................................
3740_1_a   ..........................................................................
3740_1_a   ..........................................................................
3740_1_a   ..........................................................................
3740_1_b   ...........T..............................................................
3740_1_b   ..........................................................................
3740_1_b   ..........................................................................
3740_1_b   ..........................................................................
3740_1_b   ..........................................................................
3740_2_a   ..........................................................................
3740_2_a   ..........................................................................
3740_2_a   ..........................................................................
3740_2_a   ..........................................................................
3740_2_a   ..........................................................................
3740_2_b   ..........................................................................
3740_2_b   ..........................................................................
3740_2_b   ........................................T.................................
```

```
3740_2_b      ..........................................................................
3740_2_b      ..........................................................................
3740_2_b      ..........................................................................

consensus:
3740          TTGCGCACAAACATACAAATATGTGACCCCAAAAATTTAACCATTGAAAACCAAAAAATCTAATATACTATAAC



              11111111111111111111111111111111111111111111111111111111111111111111111111
              55555555555555555555555555555555555555555555555555555555555555555555555555
              55555555555555555555555555555555555555555555555555555555555555555555555555
              22222222223333333333444444444455555555556666666666777777777788888888889999
              01234567890123456789012345678901234567890123456789012345678901234567890123

ref           TTGCGCACAAACATACAAATATGTGACCCCAAAAATTTTACCATTGAAAACCAAAAAATCTAATATACTATAAC

3810_1_a      .......................C.................T...............-.........T....G.
3810_1_a      ....A..................C.................................-.........T....G.
3810_1_a      .......................C.................................-.........T....G.
3810_1_a      ..........................................................................
3810_1_a      .....T.................C.................................-.........T....G.
3810_1_a      .......................C.................................-.........T....G.
3810_1_a      .......................C.................................-.........T....G.
3810_1_a      .......................C.................................-.........T....G.
3810_1_b      .......................C.................................-.........T....G.
3810_1_b      .......................C.................................-.........T....G.
3810_1_b      .......................C.................................-.........T....G.
3810_1_b      .......................C.................................-.........T....G.
3810_1_b      .......................C.................................-.........T....G.
3810_1_b      .......................C.................................-.........T....G.
3810_2_a      .......................C.......................C.........-.........T....G.
3810_2_a      .......................C.................................-.........T....G.
3810_2_a      .......................C.................................-.........T....G.
3810_2_a      .......................C.................................-.........T....G.
3810_2_a      .......................C.................................-.........T....G.
3810_2_a      .......................C.................................-.........T....G.
3810_2_a      .......................C.................................-.........T....G.
3810_2_b      .......................C.................................-.........T....G.
3810_2_b      .......................C.................................-.........T....G.
3810_2_b      .......................C.................................-.........T....G.
3810_2_b      .......................C.................................-.........T....G.
3810_2_b      .......................C.................................-.........T....G.

consensus:
3810          TTGCGCACAAACATACAAATATGCGACCCCAAAAATTTAACCATTGAAAACCAAAAA-TCTAATATATTATAGC



              11111111111111111111111111111111111111111111111111111111111111111111111111
              55555555555555555555555555555555555555555555555555555555555555555555555555
              55555555555555555555555555555555555555555555555555555555555555555555555555
              22222222223333333333444444444455555555556666666666777777777788888888889999
              01234567890123456789012345678901234567890123456789012345678901234567890123

ref           TTGCGCACAAACATACAAATATGTGACCCCAAAAATTTTACCATTGAAAACCAAAAAATCTAATATACTATAAC

4519_1_a      ......................................T...................................
4519_1_a      ......................................T...................................
4519_1_a      ......................................T...................................
4519_1_a      ......................................T...................................
4519_1_a      ......................................T...................................
4519_1_b      ......................................T...................................
4519_1_b      ......................................T...................................
4519_1_b      ......................................T...................................
4519_1_b      ......................................T...................................
4519_1_b      ......................................T...................................
4519_2_a      ......................................T...................................
4519_2_a      ......................................T...................................
4519_2_a      ......................................T...................................
4519_2_a      ......................................T...................................
4519_2_a      ......................................T...................................
4519_2_a      ......................................T...................................
4519_2_b      ......................................T...................................
4519_2_b      ......................................T...................................
4519_2_b      ......................................T...................................
```

```
4519_2_b   ......................................T...................................
4519_2_b   ......................................T...................................
4519_2_b   ......................................T...................................

consensus:
4519       TTGCGCACAAACATACAAATATGTGACCCCAAAAATTTTACCATTGAAAACCAAAAAATCTAATATACTATAAC



           11111111111111111111111111111111111111111111111111111111111111111111111111
           55555555555555555555555555555555555555555555555555555555555555555555555555
           55555555555555555555555555555555555555555555555555555555555555555555555555
           22222222223333333333444444444455555555556666666666777777777788888888889999
           01234567890123456789012345678901234567890123456789012345678901234567890123

ref        TTGCGCACAAACATACAAATATGTGACCCCAAAAATTTTACCATTGAAAACCAAAAAATCTAATATACTATAAC

3377_1_a   ......................................T...................................
3377_1_a   ......................................T...................................
3377_1_a   ......................................T...................................
3377_1_a   ......................................T...................................
3377_1_a   ......................................T...................................
3377_1_a   ......................................T...................................
3377_1_b   ......................................T...................................
3377_1_b   ......................................T...................................
3377_1_b   ......................................T...................................
3377_1_b   ......................................T...................................
3377_1_b   ......................................T...................................
3377_2_a   ......................................T...................................
3377_2_a   ......................................T...................................
3377_2_a   ......................................T...................................
3377_2_a   ......................................T...................................
3377_2_a   ......................................T...................................
3377_2_a   ......................................T...................................
3377_2_b   ......................................T...................................
3377_2_b   ......................................T...................................
3377_2_b   ......................................T...................................
3377_2_b   ......................................T...................................
3377_2_b   ......................................T...................................
3377_2_b   ......................................T...................................

consensus:
3377       TTGCGCACAAACATACAAATATGTGACCCCAAAAATTTTACCATTGAAAACCAAAAAATCTAATATACTATAAC



           11111111111111111111111111111111111111111111111111111111111111111111111111
           55555555555555555555555555555555555555555555555555555555555555555555555555
           55555555555555555555555555555555555555555555555555555555555555555555555555
           22222222223333333333444444444455555555556666666666777777777788888888889999
           01234567890123456789012345678901234567890123456789012345678901234567890123

ref        TTGCGCACAAACATACAAATATGTGACCCCAAAAATTTTACCATTGAAAACCAAAAAATCTAATATACTATAAC

3273_1_a   .......................C.................................-.........T....G.
3273_1_a   .......................C.................................-.........T....G.
3273_1_a   .......................C.................................-.........T....G.
3273_1_a   .......................C.................................-.........T....G.
3273_1_a   .......................C.................................-.........T....G.
3273_1_a   .......................C.....T...........................-.........T....G.
3273_1_b   .......................C.................................-.........T....G.
3273_1_b   .......................C.................................-.........T....G.
3273_1_b   .......................C.................................-.........T....G.
3273_1_b   .......................C.................................-.........T....G.
3273_1_b   .......................C.................................-.........T....G.
3273_2_a   .......................C.................................-.........T....G.
3273_2_a   .......................C.................................-.........T....G.
3273_2_a   .......................C.................................-.........T....G.
3273_2_a   .......................C.................................-.........T....G.
3273_2_a   .......................C.................................-.........T....G.
3273_2_a   .......................C.................................-.........T....G.
3273_2_b   .......................C.................................-.........T....G.
3273_2_b   .......................C.................................-.........T....G.
3273_2_b   .......................C.................................-.........T....G.
3273_2_b   .......................C.................................-.........T....G.
3273_2_b   .......................C.................................-.........T....G.
```

```
3273_2_b    .......................C.....T...........................-.........T....G.

consensus:
3273        TTGCGCACAAACATACAAATATGCGACCCCAAAAATTTAACCATTGAAAACCAAAAA-TCTAATATATTATAGC



            11111111111111111111111111111111111111111111111111111111111111111111111111
            55555555555555555555555555555555555555555555555555555555555555555555555555
            55555555555555555555555555555555555555555555555555555555555555555555555555
            22222222223333333333444444444455555555556666666666777777777788888888889999
            01234567890123456789012345678901234567890123456789012345678901234567890123

ref         TTGCGCACAAACATACAAATATGTGACCCCAAAAATTTTACCATTGAAAACCAAAAAATCTAATATACTATAAC

2806_1_a    ..........................................................................
2806_1_a    ..........................................................................
2806_1_a    ..........................................................................
2806_1_a    ..........................................................................
2806_1_a    ..........................................................................
2806_1_a    ....A.....................................................................
2806_1_b    ..........................................................................
2806_1_b    ..........................................................................
2806_1_b    ..........................................................................
2806_1_b    ..........................................................................
2806_1_b    ..........................................................................
2806_2_a    ..........................................................................
2806_2_a    ..........................................................................
2806_2_a    ..........................................................................
2806_2_a    ..........................................................................
2806_2_a    ..........................................................................
2806_2_a    ..........................................................................
2806_2_a    ..........................................................................
2806_2_b    ..........................................................................
2806_2_b    ..........................................................................
2806_2_b    ..........................................................................
2806_2_b    ..........................................................................
2806_2_b    ..........................................................................

consensus:
2806        TTGCGCACAAACATACAAATATGTGACCCCAAAAATTTAACCATTGAAAACCAAAAAATCTAATATACTATAAC



            11111111111111111111111111111111111111111111111111111111111111111111111111
            55555555555555555555555555555555555555555555555555555555555555555555555555
            55555555555555555555555555555555555555555555555555555555555555555555555555
            22222222223333333333444444444455555555556666666666777777777788888888889999
            01234567890123456789012345678901234567890123456789012345678901234567890123

ref         TTGCGCACAAACATACAAATATGTGACCCCAAAAATTTTACCATTGAAAACCAAAAAATCTAATATACTATAAC

3310_1_a    .......................C.................................-.........T....G.
3310_1_a    .......................C.................................-.........T....G.
3310_1_a    .......................C.................................-.........T....G.
3310_1_a    .......................C.................................-.........T....G.
3310_1_a    .......................C.................................-.........T....G.
3310_1_a    .......................C.G...............................-.........T....G.
3310_1_b    .......................C.................................-.........T....G.
3310_1_b    .......................C.................................-.........T....G.
3310_1_b    .......................C.................................-.........T....G.
3310_1_b    .......................C.................................-.........T....G.
3310_1_b    .......................C.................................-.........T....G.
3310_2_a    .......................C.................................-.........T....G.
3310_2_a    .......................C.................................-.........T....G.
3310_2_a    .......................C.................................-.........T....G.
3310_2_a    .......................C.................................-.........T....G.
3310_2_a    .......................C.................................-.........T....G.
3310_2_b    .......................C.................................-.........T....G.
3310_2_b    .......................C.................................-.........T....G.
3310_2_b    .......................C.................................-.........T....G.
3310_2_b    .......................C.................................-.........T....G.
3310_2_b    .......................C.................................-.........T....G.

consensus:
3310        TTGCGCACAAACATACAAATATGCGACCCCAAAAATTTAACCATTGAAAACCAAAAA-TCTAATATATTATAGC
```

```
          11111111111111111111111111111111111111111111111111111111111111111111111111
          55555555555555555555555555555555555555555555555555555555555555555555555555
          55555555555555555555555555555555555555555555555555555555555555555555555555
          22222222223333333333444444444455555555556666666666777777777788888888889999
          01234567890123456789012345678901234567890123456789012345678901234567890123

ref       TTGCGCACAAACATACAAATATGTGACCCCAAAAATTTTACCATTGAAAACCAAAAAATCTAATATACTATAAC

4374_1_a  .....................C.C.................................-.........T....G.
4374_1_a  .......................C.................................-.........T....G.
4374_1_a  .......................C.................................-.........T....G.
4374_1_a  .......................C.................................-.........T....G.
4374_1_a  .......................C.................................-.........T....G.
4374_1_a  .......................C.................................-.........T....G.
4374_1_b  .......................C.................................-.........T....G.
4374_1_b  .......................C.................................-.........T....G.
4374_1_b  .......................C.................................-.........T....G.
4374_1_b  .......................C.................................-.........T....G.
4374_1_b  .......................C.................................-.........T....G.
4374_2_a  .............C.........C.................................-.........T....G.
4374_2_a  .......................C.................................-.........T....G.
4374_2_a  .......................C.................................-.........T....G.
4374_2_a  .....................C.C...............G.................-.........T....G.
4374_2_a  .......................C.................................-.........T....G.
4374_2_b  .......................C.................................-.........T....G.
4374_2_b  .......................C.................................-.........T....G.
4374_2_b  .......................C.................................-.........T....G.
4374_2_b  .......................C.................................-.........T....G.
4374_2_b  .......................C.................................-.........T....G.

consensus:
4374      TTGCGCACAAACATACAAATATGCGACCCCAAAAATTTAACCATTGAAAACCAAAAA-TCTAATATATTATAGC


          11111111111111111111111111111111111111111111111111111111111111111111111111
          55555555555555555555555555555555555555555555555555555555555555555555555555
          55555555555555555555555555555555555555555555555555555555555555555555555555
          22222222223333333333444444444455555555556666666666777777777788888888889999
          01234567890123456789012345678901234567890123456789012345678901234567890123

ref       TTGCGCACAAACATACAAATATGTGACCCCAAAAATTTTACCATTGAAAACCAAAAAATCTAATATACTATAAC

2095_1_a  ....A.....................................G........................T....G.
2095_1_a  ....A.....................................G........................T....G.
2095_1_a  C...A.....................................G........................T....G.
2095_1_a  ....A.....................................G........................T....G.
2095_1_a  ....A.....................................G........................T....G.
2095_1_a  ....A.....................................G........................T....G.
2095_1_b  ....A.....................................G........................T....G.
2095_1_b  ....A.....................................G........................T....G.
2095_1_b  ....A.....................................G........................T....G.
2095_1_b  ....A.....................................G........................T....G.
2095_1_b  ....A.....................................G........................T....G.
2095_2_a  ....A.....................................G........................T....G.
2095_2_a  ....A.....................................G........................T....G.
2095_2_a  ....A.....................................G........................T....G.
2095_2_a  ....A.....................................G........................T....G.
2095_2_a  ....A.....................................G........................T....G.
2095_2_b  ....A.....................................G........................T....G.
2095_2_b  ....A.....................................G........................T....G.
2095_2_b  ....A.....................................G........................T....G.
2095_2_b  ....A.....................................G........................T....G.
2095_2_b  ....A.....................................G........................T....G.

consensus:
2095      TTGCACACAAACATACAAATATGTGACCCCAAAAATTTAACCGTTGAAAACCAAAAAATCTAATATATTATAGC


          11111111111111111111111111111111111111111111111111111111111111111111111111
          55555555555555555555555555555555555555555555555555555555555555555555555555
          55555555555555555555555555555555555555555555555555555555555555555555555555
```

```
          22222222223333333333444444444455555555556666666666777777777788888888889999
          01234567890123456789012345678901234567890123456789012345678901234567890123

ref       TTGCGCACAAACATACAAATATGTGACCCCAAAAATTTTACCATTGAAAACCAAAAAATCTAATATACTATAAC

2318_1_a  ....A....................-................GC.....C..................T...G.
2318_1_a  ....A.....................................G.........................T...G.
2318_1_a  ....A.....................................G.........................T...G.
2318_1_a  ....A.....................................G.........................T...G.
2318_1_a  ....A...................................TTG.........................T...G.
2318_1_b  ....A.....................................G.........................T...G.
2318_1_b  ....A.....................................G.........................T...G.
2318_1_b  ....A.....................................G.........................T...G.
2318_1_b  ....A.....................................G.........................T...G.
2318_1_b  ....A.....................................G.........................T...G.
2318_2_a  ....A.....................................G.........................T...G.
2318_2_a  ....A.....................................G.........................T...G.
2318_2_a  ....A.....................................G.........................T...G.
2318_2_a  ....A.....................................G.........................T...G.
2318_2_a  ....A.....................................G.........................T...G.
2318_2_b  ....A.....................................G.........................T...G.
2318_2_b  ....A.....................................G.........................T...G.
2318_2_b  ....A.....................................G.........................T...G.
2318_2_b  ....A.....................................G.........................T...G.
2318_2_b  ....A.....................................G.........................T...G.

consensus:
2318      TTGCACACAAACATACAAATATGTGACCCCAAAAATTTAACCGTTGAAAACCAAAAAATCTAATATATTATAGC


          11111111111111111111111111111111111111111111111111111111111111111111111111
          55555555555555555555555555555555555555555555555555555555555555555555555555
          55555555555555555555555555555555555555555555555555555555555555555555555555
          22222222223333333333444444444455555555556666666666777777777788888888889999
          01234567890123456789012345678901234567890123456789012345678901234567890123

ref       TTGCGCACAAACATACAAATATGTGACCCCAAAAATTTTACCATTGAAAACCAAAAAATCTAATATACTATAAC

2338_1_a  ....A.....................................G.........................T...G.
2338_1_a  ....A.....................................G.........................T...G.
2338_1_a  ....A.....................................G.........................T...G.
2338_1_a  ....A.....................................G.........................T...G.
2338_1_a  ....A.....................................G.........................T...G.
2338_1_b  ....A.....................................G.........................T...G.
2338_1_b  ....A.....................................G.........................T...G.
2338_1_b  ....A.....................................G.........................T...G.
2338_1_b  ....A.....................................G.........................T...G.
2338_1_b  ..........................................G.........................T...G.
2338_2_a  ..........................................G.........................T...G.
2338_2_a  ....A.....................................G.........................T...G.
2338_2_a  ....A.....................................G.........................T...G.
2338_2_a  ....A.....................................G.........................T...G.
2338_2_a  ....A.....................................G.........................T...G.
2338_2_b  ....A.....................................G.........................T...G.
2338_2_b  ....A.....................................G.........................T...G.
2338_2_b  ....A.....................................G.........................T...G.
2338_2_b  ....A.....................................G.........................T...G.
2338_2_b  ....A.....................................G.........................T...G.

consensus:
2338      TTGCACACAAACATACAAATATGTGACCCCAAAAATTTAACCGTTGAAAACCAAAAAATCTAATATATTATAGC


          11111111111111111111111111111111111111111111111111111111111111111111111111
          55555555555555555555555555555555555555555555555555555555555555555555555555
          55555555555555555555555555555555555555555555555555555555555555555555555555
          22222222223333333333444444444455555555556666666666777777777788888888889999
          01234567890123456789012345678901234567890123456789012345678901234567890123

ref       TTGCGCACAAACATACAAATATGTGACCCCAAAAATTTTACCATTGAAAACCAAAAAATCTAATATACTATAAC

1941_1_a  ....A.....................................G.........................T...G.
1941_1_a  ....A.....................................G.........................T...GT
1941_1_a  ....A.....................................G.........................T...GT
```

```
1941_1_a    ....A.....................................G........................T....GT
1941_1_a    ....A.....................................G........................T....G.
1941_1_b    ....A.....................................G........................T....G.
1941_1_b    ....A.....................................G........................T....G.
1941_1_b    ....A.....................................G........................T....G.
1941_1_b    ....A.....................................G........................T....G.
1941_1_b    ....A.....................................G........................T....G.
1941_2_a    ....A.....................................G........................T....G.
1941_2_a    ....A.....................................G........................T....G.
1941_2_a    ....A.....................................G........................T....G.
1941_2_a    ....A.....................................G........................T....G.
1941_2_a    ....A.....................................G........................T....G.
1941_2_b    ....A.....................................G........................T....G.
1941_2_b    ....A.....................................G........................T....G.
1941_2_b    ....A.....................................G........................T....G.
1941_2_b    ....A.....................................G........................T....G.
1941_2_b    ....A.....................................G........................T....G.
1941_2_b    ....A.....................................G........................T....G.

consensus:
1941        TTGCACACAAACATACAAATATGTGACCCCAAAAATTTAACCGTTGAAAACCAAAAAATCTAATATATTATAGC



            11111111111111111111111111111111111111111111111111111111111111111111111111
            55555555555555555555555555555555555555555555555555555555555555555555555555
            55555555555555555555555555555555555555555555555555555555555555555555555555
            22222222223333333333444444444455555555556666666666777777777788888888889999
            01234567890123456789012345678901234567890123456789012345678901234567890123

ref         TTGCGCACAAACATACAAATATGTGACCCCAAAAATTTTACCATTGAAAACCAAAAAATCTAATATACTATAAC

1938_1_a    ....A.....................................G........................T....G.
1938_1_a    ....A.....................................G........................T....G.
1938_1_a    ....A.....................................G........................T....G.
1938_1_a    ....A.....................................G........................T....G.
1938_1_a    ....A.....................................G........................T....G.
1938_1_b    ....A.....................................G........................T....G.
1938_1_b    ....A.....................................G........................T....G.
1938_1_b    ....A.....................................G........................T....G.
1938_1_b    ....A.....................................G........................T....G.
1938_1_b    ....A.....................................G........................T....G.
1938_2_a    ....A.....................................G........................T......
1938_2_a    ....A.....................................G........................T....G.
1938_2_a    ....A.....................................G........................T......
1938_2_a    ....A.....................................G........................T....G.
1938_2_a    ....A.....................................G........................T....G.
1938_2_b    ....A.....................................G........................T....G.
1938_2_b    ....A.....................................G........................T....G.
1938_2_b    ....A.....................................G........................T....G.
1938_2_b    ....A.....................................G........................T....G.
1938_2_b    ....A.....................................G........................T....G.

consensus:
1938        TTGCACACAAACATACAAATATATGACCCCAAAAATTTAACCGTTGAAAACCAAAAAATCTAATATATTATAGC



            11111111111111111111111111111111111111111111111111 111111111111111111111111
            55555555555555555555555555555555555555555555555555 555555555555555555555555
            55555555555555555555555555555555555555555555555555 555555555555555555555555
            22222222223333333333444444444455555555556666666666 777777777788888888889999
            01234567890123456789012345678901234567890123456789 012345678901234567890123

ref         TTGCGCACAAACATACAAATATGTGACCCCAAAAATTTTACCATTGAAAA CCAAAAAATCTAATATACTATAAC

2222_1_a    ....A.....................................G.......-.................T....G.
2222_1_a    ....A.....................................G.......-.................T....G.
2222_1_a    ....A.....................................G.......C.................T....G.
2222_1_a    ....A.....................................G.......-.................T....G.
2222_1_a    ....A.....................................G.......-.........T.......T....G.
2222_1_b    ....A.....................................G.......-.................T....GT
2222_1_b    ....A.....................................G.......-.........T.......T....G.
2222_1_b    ....A.....................................G.......-.................T....G.
2222_1_b    ....A.....................................G.......-.................T....G.
2222_1_b    ....A.....................................G.......-.................T....G.
```

```
2222_2_a    ....A.....................................G.......-.................T....G.
2222_2_a    ....A.....................................G.......-.................T....G.
2222_2_a    ....A.....................................G.......-.................T....GT
2222_2_a    ....A.....................................G.......-.................T....G.
2222_2_a    ....A.....................................G.......-.................T....G.
2222_2_b    ....A.....................................G.......-.................T....G.
2222_2_b    ....A.....................................G.......-.................T....G.
2222_2_b    ....A.................A...................G.......-.................T....G.
2222_2_b    ....A.....................................G.......-.................T....G.
2222_2_b    ....A.....................................G.......-.................T....G.

consensus:
2222        TTGCACACAAACATACAAATATGTGACCCCAAAAATTTAACCGTTGAAAA-CCAAAAAATCTAATATATTATAGC



            11111111111111111111111111111111111111111111111111111111111111111111111111
            55555555555555555555555555555555555555555555555555555555555555555555555555
            55555555555555555555555555555555555555555555555555555555555555555555555555
            22222222223333333333444444444455555555556666666666777777777788888888889999
            01234567890123456789012345678901234567890123456789012345678901234567890123

ref         TTGCGCACAAACATACAAATATGTGACCCCAAAAATTTTACCATTGAAAACCAAAAAATCTAATATACTATAAC

1093_1_a    ....A.................................GT..G........................T....G.
1093_1_a    ....A.....................................G........................T....G.
1093_1_a    ....G.....................................................................
1093_1_a    ....A.....................................G........................T....G.
1093_1_a    ....A.....................................G........................T....G.
1093_1_b    ....A.....................................G........................T....G.
1093_1_b    ....A.....................................G........................T....G.
1093_1_b    ....A.....................................G........................T....G.
1093_1_b    ....A.....................................G........................T....G.
1093_1_b    ....A.....................................G........................T....G.
1093_2_a    ....A.....................................G........................T....G.
1093_2_a    ....A.....................................G........................T....G.
1093_2_a    ....A.....................................G........................T....G.
1093_2_a    ....A.....................................G........................T....G.
1093_2_a    ....A.....................................G........................T....G.
1093_2_b    ....A.....................................G........................T....G.
1093_2_b    ....A.....................................G........................T....G.
1093_2_b    ....A.....................................G........................T....G.
1093_2_b    ....A.....................................G........................T....G.
1093_2_b    ....A.....................................G........................T....G.

consensus:
1093        TTGCACACAAACATACAAATATGTGACCCCAAAAATTTAACCGTTGAAAACCAAAAAATCTAATATATTATAGC



            11111111111111111111111111111111111111111111111111111111111111111111111111
            55555555555555555555555555555555555555555555555555555555555555555555555555
            55555555555555555555555555555555555555555555555555555555555555555555555555
            22222222223333333333444444444455555555556666666666777777777788888888889999
            01234567890123456789012345678901234567890123456789012345678901234567890123

ref         TTGCGCACAAACATACAAATATGTGACCCCAAAAATTTTACCATTGAAAACCAAAAAATCTAATATACTATAAC

4793_1_a    ....A.....................................G........................T....G.
4793_1_a    ....A.......................T......................................T....G.
4793_1_a    ....A.....................................G........................T....G.
4793_1_a    ....A.....................................G........................T....G.
4793_1_a    ....A.....................................G........................T....G.
4793_1_b    ....A.....................................G........................T....G.
4793_1_b    ....A.....................................G........................T....G.
4793_1_b    ....A.....................................G........................T....G.
4793_1_b    ....A.....................................G........................T....G.
4793_1_b    ....A.....................................G........................T....G.
4793_2_a    ....A.....................................G........................T....G.
4793_2_a    ....A.....................................G........................T....G.
4793_2_a    ....A.....................................G........................T....G.
4793_2_a    ....A.....................................G........................T....G.
4793_2_a    ....A.....................................G........................T....G.
4793_2_b    ....A.....................................G........................T....G.
4793_2_b    ....A.....................................G........................T....G.
4793_2_b    ....A.....................................G........................T....G.
```

```
4793_2_b    ....A.....................................G........................T....G.
4793_2_b    ....A.....................................G........................T....G.

consensus:
4793        TTGCACACAAACATACAAATATGTGACCCCAAAAATTTAACCGTTGAAAACCAAAAAATCTAATATATTATAGC


            11111111111111111111111111111111111111111111111111111111111111111111111111
            55555555555555555555555555555555555555555555555555555555555555555555555555
            55555555555555555555555555555555555555555555555555555555555555555555555555
            22222222223333333333444444444455555555556666666666777777777788888888889999
            01234567890123456789012345678901234567890123456789012345678901234567890123

ref         TTGCGCACAAACATACAAATATGTGACCCCAAAAATTTTACCATTGAAAACCAAAAAATCTAATATACTATAAC

256_1_a     ......................................T...................................
256_1_a     ......................................T.................................G.
256_1_a     ......................................T...........A.......................
256_1_a     ......................................T...................................
256_1_a     ......................................T...................................
256_1_b     ......................................T...................................
256_1_b     ......................................T...................................
256_1_b     ......................................T...................................
256_1_b     ......................................T...................................
256_1_b     ......................................T...................................
256_2_a     ......................................T...................................
256_2_a     ......................................T...................................
256_2_a     ......................................T...................................
256_2_a     ......................................T...................................
256_2_a     ......................................T...................................
256_2_b     ......................................T...................................
256_2_b     ......................................T...................................
256_2_b     ......................................T...................................
256_2_b     ......................................T...................................
256_2_b     ......................................T...................................

consensus:
256         TTGCGCACAAACATACAAATATGTGACCCCAAAAATTTTACCATTGAAAACCAAAAAATCTAATATACTATAAC


            11111111111111111111111111111111111111111111111111111111111111111111111111
            55555555555555555555555555555555555555555555555555555555555555555555555555
            55555555555555555555555555555555555555555555555555555555555555555555555555
            22222222223333333333444444444455555555556666666666777777777788888888889999
            01234567890123456789012345678901234567890123456789012345678901234567890123

ref         TTGCGCACAAACATACAAATATGTGACCCCAAAAATTTTACCATTGAAAACCAAAAAATCTAATATACTATAAC

5_1_a       ....A.....................................G........................T....G.
5_1_a       ....A.....................................G........................T....G.
5_1_a       ....A.....................................G........................T....G.
5_1_a       ....A..................................G.TT........................T...GC.
5_1_a       ....A.....................................G........................T....GT
5_1_b       ....A.................-...................G........................T....G.
5_1_b       ....A.....................................G........................T....G.
5_1_b       ....A.....................................G........................T....GT
5_1_b       ....A.....................................G........................T....GT
5_1_b       ....A.....................................G........................T....G.
5_2_a       ....A.....................................G........................T....G.
5_2_a       ....A.....................................G........................T....G.
5_2_a       ....A.....................................G........................T....G.
5_2_a       ....A.....................................T........................T....G.
5_2_a       ....A.....................................G........................T....G.
5_2_b       ....A.....................................G........................T....G.
5_2_b       ....A.....................................G........................T....G.
5_2_b       ....A.....................................G........................T....G.
5_2_b       ....A.....................................G........................T....G.
5_2_b       ....A.....................................G........................T....G.

consensus:
5           TTGCACACAAACATACAAATATGTGACCCCAAAAATTTAACCGTTGAAAACCAAAAAATCTAATATATTATAGC
```

```
          11111111111111111111111111111111111111111111111111111111111111111111111111
          55555555555555555555555555555555555555555555555555555555555555555555555555
          55555555555555555555555555555555555555555555555555555555555555555555555555
          22222222223333333333444444444455555555556666666666777777777788888888889999
          01234567890123456789012345678901234567890123456789012345678901234567890123

ref       TTGCGCACAAACATACAAATATGTGACCCCAAAAATTTTACCATTGAAAACCAAAAAATCTAATATACTATAAC

920_1_a   ....A.....................................G........................T....G.
920_1_a   ....A.....................................G........................T....G.
920_1_a   ....A.....................................G........................T....G.
920_1_a   ....A.....................................G........................T....GT
920_1_a   ....A.....................................G........................T....GT
920_1_a   ....A.....................................G........................T....G.
920_1_b   ....A.....................................G........................T....G.
920_1_b   ....A.....................................G........................T....G.
920_1_b   ....A.....................................G........................T....G.
920_1_b   ....A.....................................G........................T....G.
920_1_b   ....A.....................................G........................T....G.
920_2_a   ....A.....................................G........................T....G.
920_2_a   ....A.....................................G........................T....G.
920_2_a   ....A.....................................G........................T....G.
920_2_a   ....A.....................................G........................T....G.
920_2_a   ....A.....................................G........................T....G.
920_2_b   ....A.....................................G........................T....G.
920_2_b   ....A.....................................G........................T....G.
920_2_b   ....A.....................................G........................T....G.
920_2_b   ....A.....................................G........................T....G.
920_2_b   ....A.....................................G........................T....G.

consensus:
920       TTGCACACAAACATACAAATATGTGACCCCAAAAATTTAACCGTTGAAAACCAAAAAATCTAATATATTATAGC


          11111111111111111111111111111111111111111111111111111111111111111111111111
          55555555555555555555555555555555555555555555555555555555555555555555555555
          55555555555555555555555555555555555555555555555555555555555555555555555555
          22222222223333333333444444444455555555556666666666777777777788888888889999
          01234567890123456789012345678901234567890123456789012345678901234567890123

ref       TTGCGCACAAACATACAAATATGTGACCCCAAAAATTTTACCATTGAAAACCAAAAAATCTAATATACTATAAC

1138_1_a  ....A.....................................G........................T....G.
1138_1_a  ....A.....................................G........................T....G.
1138_1_a  ....A.....................................G........................T....G.
1138_1_a  ....A.....................................G........................T....G.
1138_1_a  ....A.....................................G........................T....G.
1138_1_b  ....A.....................................G........................T....G.
1138_1_b  ....A.....................................G........................T....G.
1138_1_b  ....A.....................................G........................T....G.
1138_1_b  ....A.....................................G........................T....G.
1138_1_b  ....A.....................................G........................T....G.
1138_2_a  ....A.....................................G........................T....G.
1138_2_a  ....A.....................................G........................T....G.
1138_2_a  ....A.....................................G........................T....G.
1138_2_a  ....A.....................................G........................T....G.
1138_2_a  ....A.....................................G........................T....G.
1138_2_b  ....A.....................................G........................T....G.
1138_2_b  ....A.....................................G........................T....G.
1138_2_b  ....A.....................................G........................T....G.
1138_2_b  ....A.....................................G........................T....G.
1138_2_b  ....A.....................................G........................T....G.

consensus:

1138      TTGCACACAAACATACAAATATGTGACCCCAAAAATTTAACCGTTGAAAACCAAAAAATCTAATATATTATAGC


          11111111111111111111111111111111111111111111111111111111111111111111111111
          55555555555555555555555555555555555555555555555555555555555555555555555555
          55555555555555555555555555555555555555555555555555555555555555555555555555
          22222222223333333333444444444455555555556666666666777777777788888888889999
          01234567890123456789012345678901234567890123456789012345678901234567890123
```

```
ref          TTGCGCACAAACATACAAATATGTGACCCCAAAAATTTTACCATTGAAAACCAAAAAATCTAATATACTATAAC

195_1_a      ....A.....................................G........................T....G.
195_1_a      ....A.....................................G........................T....G.
195_1_a      ....A.....................................G........................T....G.
195_1_a      ....A.....................................G........................T....G.
195_1_a      ....A.....................................G........................T....G.
195_1_b      ....A.....................................G........................T....G.
195_1_b      ....A.....................................G........................T....G.
195_1_b      ....A.....................................G........................T....G.
195_1_b      ....A.....................................G........................T....G.
195_1_b      ....A.....................................G........................T....G.
195_2_a      ....A.....................................G........................T....G.
195_2_a      ....A.....................................G........................T....G.
195_2_a      ....A.....................................G........................T....G.
195_2_a      ....A.....................................G........................T....G.
195_2_a      ....A.....................................G........................T....G.
195_2_b      ....A.....................................G........................T....G.
195_2_b      ....A.....................................G........................T....G.
195_2_b      ....A.....................................G........................T....G.
195_2_b      ....A.....................................G........................T....G.
195_2_b      ....A.....................................G........................T....G.

consensus:
195          TTGCACACAAACATACAAATATGTGACCCCAAAAATTTAACCGTTGAAAACCAAAAAATCTAATATATTATAGC
```

```
             11111111111111111111111111111111111111111111111111111111111111111111111111
             55555555555555555555555555555555555555555555555555555555555555555555555555
             55555555555555555555555555555555555555555555555555555555555555555555555555
             22222222223333333333444444444455555555556666666666777777777788888888889999
             01234567890123456789012345678901234567890123456789012345678901234567890123

ref          TTGCGCACAAACATACAAATATGTGACCCCAAAAATTTTACCATTGAAAACCAAAAAATCTAATATACTATAAC

2986_1_a     ....A.....................................G........................T....G.
2986_1_a     ....A.....................................G........................T....G.
2986_1_a     ....A.....................................G........................T....G.
2986_1_a     ....A.....................................G........................T....G.
2986_1_a     ....A.....................................G........................T....G.
2986_1_b     ....A.....................................G........................T....G.
2986_1_b     ....A.....................................G........................T....G.
2986_1_b     ....A.....................................G........................T....G.
2986_1_b     ....A.....................................G........................T....G.
2986_1_b     ....A.....................................G........................T....G.
2986_2_a     ....A.....................................G........................T....G.
2986_2_a     ....A.....................................G........................T....G.
2986_2_a     ....A.....................................G........................T....G.
2986_2_a     ....A.....................................G........................T....G.
2986_2_a     ....A.....................................G........................T....G.
2986_2_b     ....A.....................................G........................T....G.
2986_2_b     ....A.....................................G........................T....G.
2986_2_b     ....A.....................................G........................T....G.
2986_2_b     ....A.....................................G........................T....G.
2986_2_b     ....A.....................................G........................T....G.

consensus:
2986         TTGCACACAAACATACAAATATGTGACCCCAAAAATTTAACCGTTGAAAACCAAAAAATCTAATATATTATAGC
```

```
             11111111111111111111111111111111111111111111111111111111111111111111111111
             55555555555555555555555555555555555555555555555555555555555555555555555555
             55555555555555555555555555555555555555555555555555555555555555555555555555
             22222222223333333333444444444455555555556666666666777777777788888888889999
             01234567890123456789012345678901234567890123456789012345678901234567890123

ref          TTGCGCACAAACATACAAATATGTGACCCCAAAAATTTTACCATTGAAAACCAAAAAATCTAATATACTATAAC

consensus:
5013         TTGCGCACAAACATACAAATATGCGACCCCAAAAATTTAACCATTGAAAACCAAAAA-TCTAATATATTATAGC
```

```
           11111111111111111111111111111111111111111111111111111111111111111111111111
           55555555555555555555555555555555555555555555555555555555555555555555555555
           55555555555555555555555555555555555555555555555555555555555555555555555555
           22222222223333333333444444444455555555556666666666777777777788888888889999
           01234567890123456789012345678901234567890123456789012345678901234567890123

ref        TTGCGCACAAACATACAAATATGTGACCCCAAAAATTTTACCATTGAAAACCAAAAAATCTAATATACTATAAC

consensus:
F3479      TTGCGCACAAACATACAAATATGTGACCCCAAAAATTTAACCATTGAAAACCAAAAAATCTAATATACTATAAC
```

Table S4: Codes of analyzed samples and information about US, anatomical element and age

Sample code	US	Anatomical element	Age
1	2	I^2 left	Juv
3740	3A	I^1 left bud	Juv
3810	3A	I_2 right	Sen
4519	3A	I^2 right	Juv
3377	3A	I^2 left	Juv
3273	3A	Mand+M_2 right	Ad
2806 *	3A	I_2 left	Ad
5012	3B	Mand+P_3 left	Ad
3310	3B	D_3 left	Juv
4374	3B	P_3 left	Ad
2095	4	D^4 left	Juv
2318	4	Masc+D^4+M^1 left	Juv
2320 **	4	Masc+D^4+M^1 right	Juv
2338	4	C^x left	Sen
1941	4	P^2 right	Ad
1938	4	Masc+P^2 right	Ad
2222	4	Masc+P^2 right	Ad
2106 ***	4	Mand+M_3 right	Ad
1093	5	Masc+p^4 - M^3 left	Ad
4793	5	Masc+p^{4} - M^2 left	Ad
1512	5	D_4 right	Juv
597	5	D_4 right	Juv
256	5	D_4 right	Juv
1669	5	M^1 left	Sen
5	5	Masc+P^4+M^1 left	Ad
920	5	Mand+D_4 - M_2 right	Juv
1138	5	M^1 left	Sen
921+922	5	Masc+P^{2} - P^4 left	Ad
195	5	D_4 right	Juv
1661	5	P_2 right	Juv-Ad
881	5	P_2 right	Juv-Ad
2986	5	P_2 right	Juv-Ad

* possible duplicate of 3273

** possible duplicate of 2095 or 2318

*** possible duplicate of 1941, 1938 or 2222
